# How and why beekeepers participate in the INSIGNIA citizen science honey bee environmental monitoring project

**DOI:** 10.1007/s11356-021-13379-7

**Published:** 2021-03-16

**Authors:** Kristina Gratzer, Robert Brodschneider

**Affiliations:** Institute of Biology, University of Graz, Universitätsplatz 2, Graz, 8010 Austria

**Keywords:** *Apis mellifera*, Pesticides, Pollen, Public participation, Survey, Volunteer motivation

## Abstract

In the “contributory” citizen science project INSIGNIA, beekeepers carried out non-invasive sampling of their own honey bee colonies for an environmental investigation of pesticide residues and pollen plant origin. We surveyed several traits and attitudes of 69 of the volunteering beekeepers from ten countries. We found that their motivation was similar to that found in previous studies of environmental volunteer motivation, with helping the environment and contributing to scientific knowledge being strong motivators. Our results suggest that receiving laboratory analysis results of the samples from their colonies is the most meaningful way of appreciation for beekeepers, but is not their primary reason for participation. A citizen scientist beekeeper in this study spent on average 10.4 working hours on the project during a sampling season. Our study indicates that most of our volunteers would participate in similar future investigations, or would recommend participation to other beekeepers, underlining the potential of beekeepers as citizen scientists in honey bee research.

**Supplementary Information:**

The online version contains supplementary material available at 10.1007/s11356-021-13379-7.

## Introduction

“Citizen science” is becoming more and more popular in the environmental sciences, monitoring and ecology (Dickinson et al. 2010; Miller-Rushing et al. 2012; Theobald et al. 2015; Parsons et al. 2018; MacPhail and Colla 2020). There are many definitions and forms of citizen science, but common characteristics are the recruitment and training (or instructing) of volunteers by research institutions (Bonney et al. 2009a; Heigl et al. 2019; Vohland et al. 2021). In entomology, citizen science has a long tradition, but only recently has the number of scientific articles credited as such increased. Koffler et al. (2021) present a meta-analysis of 88 research articles, which mostly deal with bumble bees and honey bees. In the majority of these studies, volunteer participation was restricted to data collection. It is important to note that not all scientific activities involving public participation are citizen science. Heigl et al. (2020) provide an overview of important quality criteria. If suitable scientific principles are followed, the findings of citizen science studies can be analysed, summarized and published in peer-reviewed journals (Gadermaier et al. 2018; Ward-Fear et al. 2020). Participation of citizens in scientific contexts has much wider societal effects. To name a few, Toomey and Domroese (2013) suggested an influence on participants’ perceptions of their conservation behaviours, while Trumbull et al. (2000) rated the thinking process of citizen scientists as similar to those in professional scientific investigations.

Beekeepers can contribute to research on honey bees, and the environment they are foraging in in several ways, ranging from providing data for crowdsourcing investigations, to helping research with sample collection (Brodschneider et al. 2019; Gray et al. 2020; Morawetz et al. 2020). One of the assets of citizen science is reaching out to plentiful, often distant and private research locations (Pocock et al. 2014). The special case with beekeepers is that one can safely assume that they have expert knowledge of honey bee husbandry. Beekeepers understand terminology, are likely to quickly adopt new findings, own livestock and have basic equipment for colony manipulations, which makes them a specialized group for honey bee-related studies. With an estimated 620,000 beekeepers in Europe (Chauzat et al. 2013), this group constitutes a huge potential resource of skilled volunteers for scientific studies. The majority of beekeepers are hobbyists harvesting honey for their own use or side income, but not as their primary source of income (Brodschneider et al. 2010; Alonso et al. 2020; Tomljanović et al. 2020). Their motivations to participate in citizen science studies and their abilities in fulfilling different tasks required for participation in environmental monitoring have, however, not yet been studied (Koffler et al. 2021). Volunteer motivation in general has been investigated manifold times in a variety of fields. Among others, general notions discussed by many authors are altruism, social interactions or care for the environment (Phillips 1982; Haski-Leventhal 2009; Measham and Barnett 2008). For citizen science, a strong motivation for volunteers is to improve their own understanding of science, and to help scientists make new discoveries (West and Pateman 2016; Curtis 2018).

Of great interest for research is the interaction of honey bees with their environment, which makes bees and bee products very suitable for biomonitoring (Herrero-Latorre et al. 2017; Smith et al. 2019). Adult honey bees, for example, have been sampled as environmental monitors and analysed to detect pollutants in the environment (Balestra et al. 1992; van der Steen et al. 2015; Gómez-Ramos et al. 2019). Recently detection of downwind lead contamination in Parisian honeys after the 2019 fire of Notre-Dame cathedral was showcased by Smith et al. (2020). In some environmental monitoring investigations, volunteer beekeepers have been involved in collecting pollen samples to study forage plant diversity or pesticides. An often used approach for this is the collection of pollen loads from returning foragers with pollen traps (Conti et al. 2016; Drummond et al. 2018; Tosi et al. 2018; Brodschneider et al. 2019).

Our study is the first to quantify several traits and attitudes of beekeepers acting as citizen scientists in a season-long environmental monitoring project. In 2019, the first year of the INSIGNIA (citizen science investigation for pesticides in apicultural products) project, a small number of beekeepers participated in four European countries. The beekeepers’ participation can be classified as a “contributory” project according to the classification of Shirk et al. (2012). The volunteers used four different non-invasive sampling methods every 2 weeks on three of their honey bee colonies. The main aim of the project was not to make use of the citizen science potential to generate a large dataset, but was to identify best practice protocols that can be applied by volunteers. The study was accompanied by detailed interviews with the citizen scientists at the beginning, and towards the end, of the first project season conducted by a social scientist. Bieszczad et al. (in prep.) provide some qualitative insights gained through this, focusing on the self-conceptualization of the citizen scientists’ roles. Based on a small, relatively homogenous sample of participants, they found a considerable diversity in how the citizen scientists conceptualize their roles, namely as amateur scientist, expert, assistant or knowledge broker. Several findings from these interviews regarding sampling protocols were integrated in the study plan for the second field season of the project.

For the 2020 field season, the number of participants was extended, but the protocol for citizen scientist beekeepers was reduced and simplified, compared to season one (Brodschneider et al. 2021). Beekeepers collected pollen using pollen traps on two of their colonies every 2 weeks, packed and labelled the pollen samples and stored them in freezers. These pollen samples were then used for ITS2 metabarcoding (Richardson et al. 2015) to determine their botanical origin and hence the plants on which the bees had been foraging. Participants also placed “APIStrips” in the broodnest of the two colonies. These strips were an innovation resulting from the first year of the INSIGNIA project, and adsorb substances on their Tenax surface (Murcia-Murales et al. 2020) and can later be analysed for pesticide residues. Additionally, beekeepers regularly estimated the number of bees in each colony, and reported the flowering of 30 selected plants in the vicinity of their test colonies. All participants received the special materials required for sample collection and storage, and a pamphlet with illustrated step-by-step explanations of all tasks. Materials that are usually available in a household or many beekeeping operations, like kitchen towels or pollen traps, were not generally provided. Communication channels between coordinators in each country and their citizen scientists were established via several different routes (email, Facebook, WhatsApp). As suggested by Bieszczad et al. (in prep.), beekeepers were promised to receive detailed laboratory analysis of the samples they collected, but the present survey was conducted at the end of the 2020 sampling season before the laboratory results of samples were available. However, at the time of the survey, citizen scientists had already accomplished eight samplings over the previous 3 months.

According to West and Pateman (2016), knowing potential volunteers’ motivations, their personal attributes, circumstances and demographics, and how they will become aware of the opportunity to participate in a study, is a key factor in the organization of a citizen science investigation. In the present article, we have studied for the first time why beekeepers from ten countries volunteer and cooperate with scientists. We electronically surveyed their opinion on several aspects of their participation in the INSIGNIA citizen science project. In this study, we report the characteristics of the beekeepers participating as citizen scientists, their reasons for volunteering, their assessment of difficulty of different tasks required for participation and colony-related problems occurring during the project. As the citizen science project dealt with environmental topics including intensively discussed possible drivers of honey bee decline (Goulson et al. 2015; Donkersley et al. 2020; Hristov et al. 2020), we asked beekeepers to rank areas where they expect impact of the project. We finally asked beekeepers about the time budget they voluntarily invested for sampling. We hope that this article expands the application of citizen science in honey bee research and helps in recruiting volunteers as well as designing studies according to the needs and skills of both beekeepers and scientists.

## Material and methods

We collected information from citizen scientists participating in the INSIGNIA project using a closed online questionnaire with personalized links in Limesurvey (version 3.22.19, Limesurvey GmbH., Hamburg, Germany). The first invitation email was sent on 12.8.2020 to the email addresses the participants used for project communication. Those recipients who did not respond were reminded on 17.8.2020, with a final reminder on 23.8.2020. The survey was available in languages of all of the countries where the citizen scientists lived (English, German, Danish, French, Greek, Italian, Latvian, Dutch; the one Norwegian participant answered the Danish version). The full questionnaire including all answer options (see S1 and S2) and the Limesurvey .lss file (S3) with questions in all languages are available as electronic supplementary material.

### Citizen scientists’ demographics

Sex, age, employment, education, years of beekeeping experience and the number of honey bee colonies owned by participants were asked. We also asked how they became aware of the opportunity to participate in the project (answer options: article in beekeeping magazine; project or other website; social media; oral presentation; personal contact with coordinators; friends). Multiple answers were possible in response to this question.

### Citizen scientists’ reason for participation

Participants were asked for their level of agreement in a Likert-type five-point response format (strongly agree, agree, neutral or undecided, disagree, strongly disagree) to statements about their reasons for volunteering for the project. These mainly followed those developed by Bruyere and Rappe (2007), Raddick et al. (2009), Asah and Blahna (2012), Nov et al. (2014) and Alender (2016), and were I want to... (a) help or enhance the environment, (b) help the community, (c) get outside or connect with nature, (d) contribute to scientific knowledge, (e) learn more about honey bees, (f) do something physically active, (g) learn skills or new knowledge, (h) have fun, (i) help the project to do more for less money, (j) engage with other people, (k) enhance my reputation in my community, (l) advance my career through gained experience or networking, (m) increase public safety and (n) receive free laboratory analysis of my samples. Item e was modified to include the honey bee as a study subject.

### Citizen scientists’ impact expectations

To address gaps in the literature, a novel question about the citizen scientists’ expectations was included. Participants were asked to rank the areas they think the research of the INSIGNIA citizen science project will most likely have an impact (rank 1) to least likely have an impact (rank 7). The areas presented in random order in the survey were politics, science, economy, agriculture, beekeeping, environment and innovation.

### Recognition and appreciation

Respondents were asked to indicate how meaningful different forms of recognition were to them on a scale from “not meaningful at all” to “very meaningful”. Expanding the items developed by Alender (2016), two forms of recognition particular to this study (the feedback on pesticide and botanical analysis of citizen scientists’ own samples) were added. The list of items therefore included the following: hand-written card; volunteer appreciation event; certificate or token of appreciation; paraphernalia (stickers, hats, t-shirts from project); name recognition in social media; name recognition in a scientific publication; results feedback on own samples (pollen diversity); results feedback on own samples (pesticide residues); individual co-authorship on scientific publication; and group co-authorship on scientific publication (“Insignia-beekeepers”).

### Difficulty assessment

The workload required for project participation was divided into 21 distinctive tasks by the study authors and beekeepers were asked to rate each individual task from 1 (very easy) to 10 (very difficult). The tasks were as follows: communication with project coordinators; understanding terminology; understanding pamphlet; understanding project aims; preparing colonies for the sampling; organizing the materials needed for the project in time; picking the best day for sampling; pollen trap usage; pollen harvest; measuring the required amount of pollen; working with the APIStrips; filling out sample labels; estimating the number of occupied beelanes; giving information on the phenology of flowering plants; answering the electronic survey; providing location using the online map; sample storage; test hive management throughout the season; retaining motivation for participation throughout season; photo documentation of collected samples; and accurate working while sampling.

### Differences among demographic groups

The survey questions on motivation, appreciation and difficulty were statistically analysed for differences among demographic groups using the following categories: (1) age (30-45, 46-60, 60+ years); (2) beekeeping experience (0-15 years, 16-30 and 30+ years); (3) sex (male vs. female); and (4) education (college vs. no college). The first two groups (with three categories) were analysed in IBM SPSS statistics 26 with Kruskal-Wallis tests and post hoc Mann-Whitney *U* tests with Holm-Bonferroni correction (Holm, 1979). The two demographic traits with only two categories were analysed with Mann-Whitney *U* tests only. For the tests, answer options (strongly agree to strongly disagree, and very meaningful to not meaningful at all) were re-coded 1-5, with “neutral or undecided” being 3. For assessment of difficulty of tasks required for project participation, volunteers’ ratings of 1 to 10 were the target variable.

### Complications experienced during the investigation

Citizen scientists were asked to report on typical problems that can occur in hive management during a sample season for their two test colonies only. These complications included the following: supersedure; swarming; colony dwindling; colony mortality; brood disease (American foulbrood and others); and replacement of the test colony with another one, or broken equipment (e.g. pollen trap). Answer options for each of the sub questions were “No”, “Yes - in one colony” and “Yes - in both colonies”.

### Workload and continuation

We asked citizen scientists to estimate the field working time for one complete sampling of both colonies (in minutes), excluding travel time. Secondly, we asked them to also estimate travel time (outward and return) to reach the test hives for sampling (in minutes). The averages of these two questions were summed and used to extrapolate workload performed by citizen scientists during the field season. As a third component, we added the average time a citizen scientist spent transmitting the data of each sampling round, as measured by the electronic survey (average of all data submissions during project duration). The sum of these three components was analysed for differences among demographic groups using the categories and statistical methodology established above. We also asked beekeepers whether they would recommend colleagues to participate in similar citizen science studies and whether they themselves would participate again in a similar citizen science investigation (answer options: definitely, very probably, probably, possibly, probably not, definitely not).

## Results

### Citizen scientists’ demographics

**Fig. 1:**
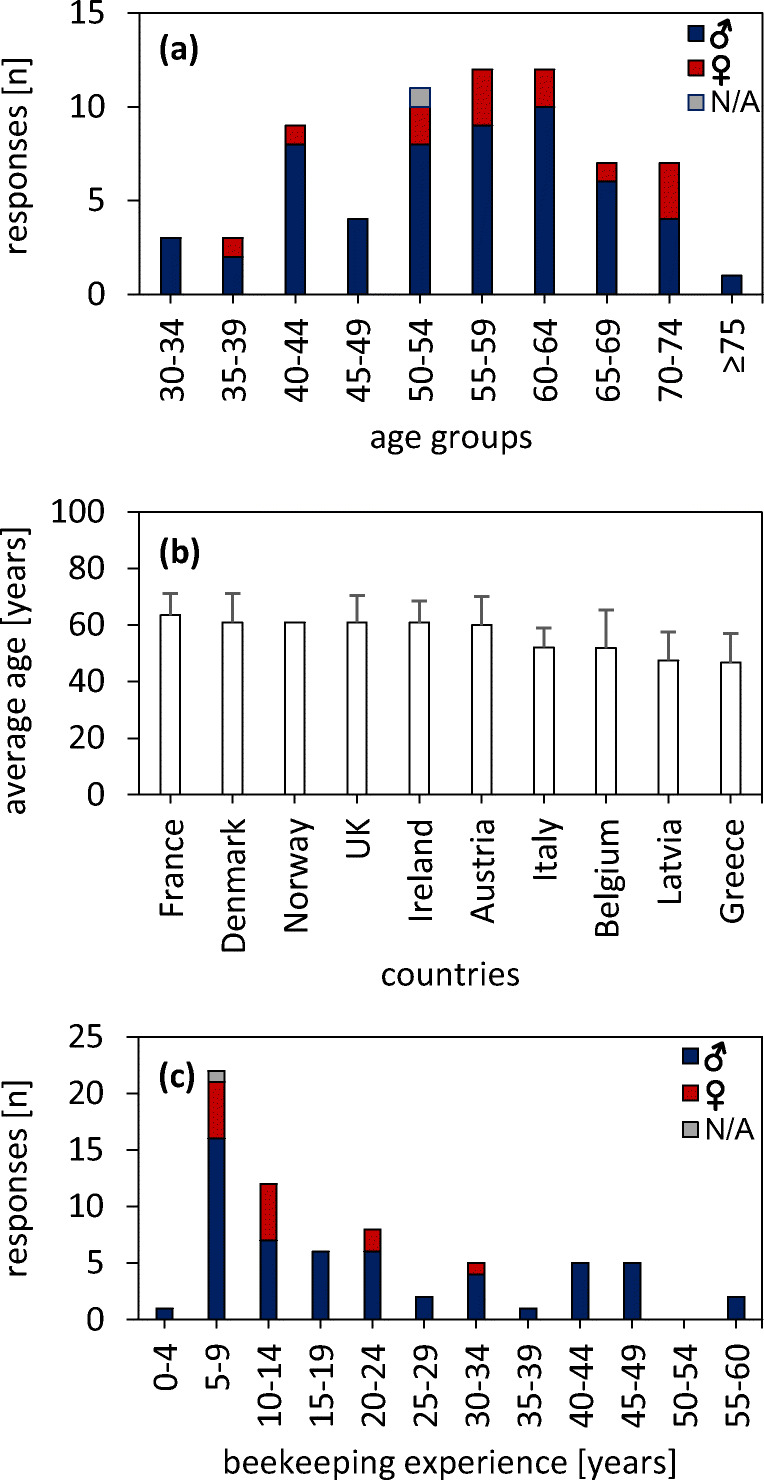
**a** Age distribution of 69 beekeepers volunteering as citizen scientists in the INSIGNIA project. **b** Average (± standard deviation) age of beekeepers in different countries (*n* = 6-9, Norway: *n* = 1). **c** Number of years of beekeeping experience of citizen scientists (*n* = 69). Blue: male, red: female, grey: N/A

Sixty-nine citizen scientists (80% male, 19% female; one participant did not disclose sex) from ten countries answered the survey. This corresponds to 89% of all invited participants. The average age was 55.4 years. The distribution of age is shown in Fig. 1a. Average age was highest (63.5 years) in France followed by Denmark and the UK. The youngest citizen scientists were in Greece (46.7) followed by Latvia and Belgium (Fig. 1b). Participating beekeepers on average managed 70.2 colonies (range: 3-320) and had a mean beekeeping experience of 19.7 years (range: 2-57, Fig. 1c).

For employment (*n* = 68), most participants were employed full time (39.1%), followed by participants being retired (29.0%), self-employed (27.5%) and part-time employed (4.3%). The question on level of education (*n* = 68) revealed that most participants had trade/technical/vocational training (33.8%), followed by a master’s degree (22.1%), high school graduate, diploma or the equivalent (19.1%) and a bachelor’s degree (19.1). A total of 5.9% of participants stated they had a doctorate degree. For further analysis (see below), the categories “no college” (*n* = 36) and “college” (*n* = 32) were formed.

Beekeepers (*n* = 69) were mostly recruited through personal contact with coordinators (71.0%) followed equally by the project website or other website, and talks at meetings (13.0%), articles in beekeeping magazines (11.6%), social media (5.8%) and friends (2.9%). Two participants additionally mentioned that they are part of the INSIGNIA consortium, one mentioned a call from their beekeeping association to participate in the project and another one is a colleague of a national coordinator. Multiple answers were possible to this question.

### Citizen scientists’ reasons for participation

**Fig. 2:**
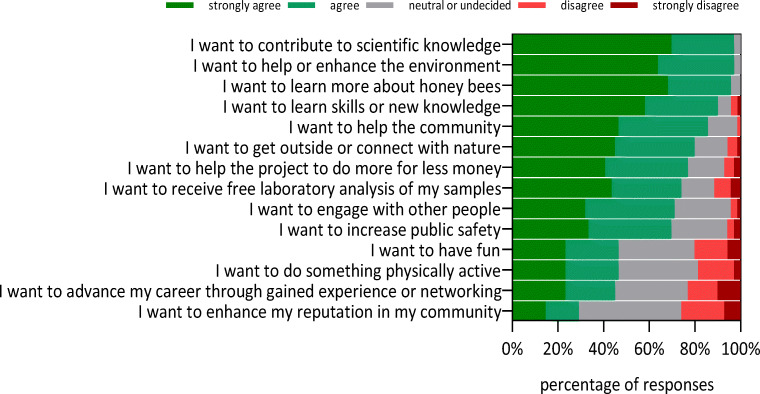
Frequency of beekeeper citizen scientists’ level of agreement with reasons for participation in order of strongest to weakest agreement. *n* = 69 each

The most common reasons for participation in the project were “to contribute to scientific knowledge” and “to help or enhance the environment” (97.1% strongly agree or agree), closely followed by “to learn more about honey bees” (95.7%) (Fig. 2). The least agreement, but highest disagreement, was given to the following reasons for participation: “to have fun”, “to do something physically active”, “to advance career” or “to enhance reputation”.

### Citizen scientists’ impact expectations

Beekeepers most commonly ranked environment, beekeeping and science as the main areas where they expect the INSIGNIA project to have impact (Table 1). They felt that economy, politics and innovation were least likely to be impacted by the project.

**Table 1:** Citizen scientist beekeepers’ ranking of their expectations on the impact of the INSIGNIA study in different areas (*n* = 69)

	Rank 1	Rank 2	Rank 3	Rank 4	Rank 5	Rank 6	Rank 7
Environment	24	20	13	6	2	4	0
Beekeeping	20	16	14	9	2	4	4
Science	18	15	13	14	6	2	1
Agriculture	3	9	17	15	15	8	2
Innovation	2	4	10	11	25	12	5
Politics	1	4	2	8	6	16	32
Economy	1	1	0	6	13	23	25

### Recognition and appreciation

**Fig. 3:**
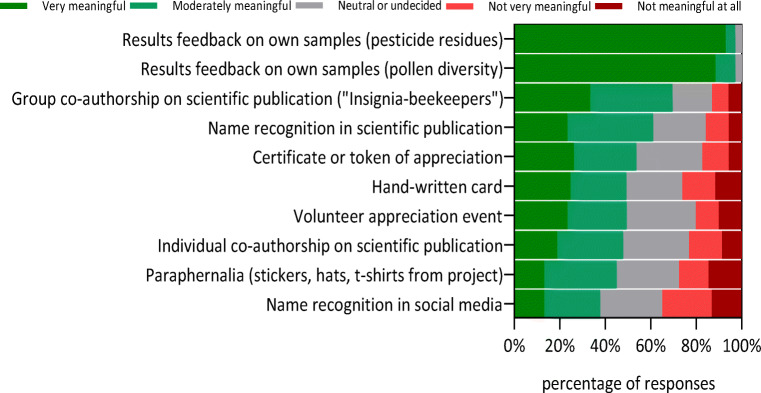
Frequency of beekeeper citizen scientists’ levels of agreement with different forms of recognition or appreciation in order of most meaningful to least meaningful. *n* = 69 each

Most citizen scientists (97.1%) rated “results feedback on own samples” for pesticide and pollen diversity as very or moderately meaningful recognition of their participation (Fig. 3). Group co-authorship of a scientific publication and name recognition in a scientific publication were rated next meaningful. Citizen scientists rated “name recognition in social media” as the least meaningful way of appreciation and 34.8% rated this as not very meaningful or not meaningful at all.

### Assessment difficulty of tasks required for project participation

**Fig. 4:**
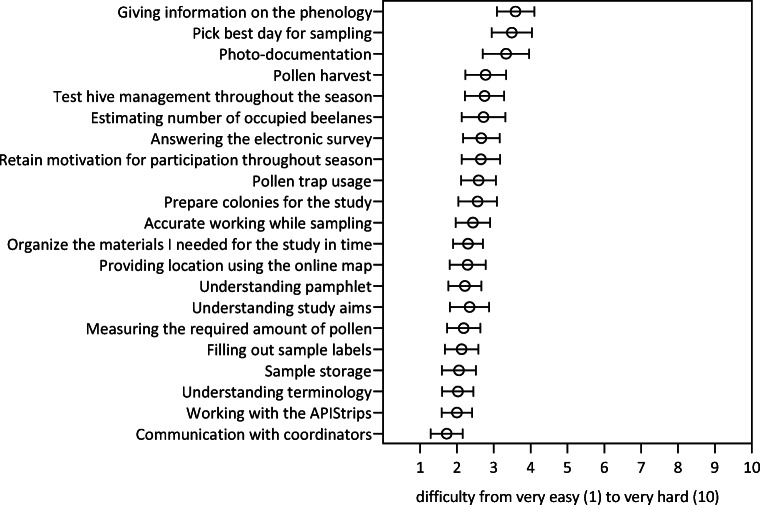
Citizen scientist beekeepers’ assessment of difficulty of different project tasks from 1 (very easy) to 10 (very difficult). Answers are presented as mean ± 95% confidence interval (*n* = 69 each)

Beekeepers assessed all required tasks on average with a difficulty rank of 2.5 (*n* = 1449), where 1 = very easy and 10 = very difficult. Of the individual tasks, they rated giving information on the phenology of flowering plants as most difficult (3.6), followed by picking the best day for sampling (3.5), and photo documentation of collected samples (3.3) (Fig. 4). Communication with project coordinators (1.7), working with APIStrips (2.0) and understanding the terminology (2.0) were rated as the simplest tasks.

### Differences between demographic groups

The significant differences between demographic groups on motivation for participating as volunteers are shown in Table S1. Participants older than 60 years agreed less with “advancing career through gained experience or networking” than the younger groups of citizen scientists. Similarly, beekeepers with 0-15 years of beekeeping experience agreed more with this claim than the two more experienced groups. The two sex-based differences were that females significantly agreed more in learning new skills and knowledge as well as in helping the environment as motivation for participation. The level of agreement for participation between citizen scientists with or without college education differed in eight investigated items, e.g. “connect with nature” or “increase public safety” (see Table S1 for full list of items). The agreement was generally higher among participants without college education. Regarding levels of agreement with different forms of recognition or appreciation, females and the middle age category agreed more that pesticide results of their own samples are a meaningful feedback (Table S2). Females significantly agreed less with paraphernalia as a form of appreciation. In the difficulty assessment of 21 different project tasks, no differences could be detected for the demographic traits age, experience and sex, but three answer options were affected by education (Table S3). College graduates had more difficulties in understanding the pamphlet, working with the APIStrips and retaining motivation for participation throughout the season.

### Complications experienced during the project

Citizen scientists were asked about whether they experienced one or more of seven common hive management complications within the field season. From 69 respondents, two-thirds experienced at least one of the following: colony dwindling (33.3% in one and 11.6% in both colonies, respectively) followed by supersedure (30.4% in one and 1.4% in both colonies); swarming (18.8% in one and 7.2% in both colonies); replacement of test colony with another one (7.2% in one and 2.9% in both colonies); equal colony mortality; and broken equipment (2.9% in one and 0% in both colonies). No respondent experienced a brood disease in the test colonies. In total, 29% reported one complication, 21.7% two, 5.8% three and 1 person dealt with five difficulties in one of the test colonies. From all respondents, 11.6% had one complication in both colonies and 5.8% reported two complications in both colonies.

### Workload and continuation

The average time spent for the field work of sampling both test colonies in one sampling round was 29.3 min (SD ± 23.7, *n* = 68). A total of 63.2% of participants reported a travel time (outward and return) greater than 5 min, while the rest had their INSIGNIA testing colonies near their homes. A maximum travel time of 120 min was reported by one participant. Average travel time of all respondents was 24.6 min (SD ± 26.8, *n* = 67).

Alongside the two self-assessments of time periods provided by citizen scientists, we determined the time citizen scientists spent submitting the data for each sampling round, measured by the electronic survey software. This was on average 8.8 min (SD ± 4.0, *n* = 768). The total time spent per sampling round per participant including all three time components therefore can be approximated to 62.6 min. Age, years of beekeeping experience, sex and education had no effect on the time budget of citizen scientists (*p* > 0.05, data not shown). Participating in the full season of ten samplings thus resulted in 10.4 h invested by each participant. Extrapolated to ten sampling events conducted by 80 beekeepers, which almost equals the number of participants in the second INSIGNIA field season, a workload of 835.2 h was accomplished by the citizen scientists in total. Based on a 40-h working week, this equates to 20.9 weeks full-time employment.

Citizen scientists mostly estimated their willingness in participating in similar citizen science studies as “definitely” (66.7%), followed by “very probably” (17.4%), “possibly” (8.7%) and “probably” (7.2%, *n* = 69). From all 69 survey respondents, the majority (65.2%) would definitely recommend colleagues to participate in a similar citizen science investigation, 20.3% very probably, 7.2% probably and 5.8% possibly recommend similar studies to others, while 1.4% would probably not recommend a participation (*n* = 69).

## Discussion

In this study, for the first time, we characterized beekeepers acting as citizen scientists. Studies about the attitudes of volunteers helping environmental research have so far been made for various general groups and highly specialized interest groups in environmental monitoring. These studies included volunteers involved in surface water quality (Alender 2016; Blake et al. 2020), hobby ornithologists (Evans et al. 2005), people helping research online (Raddick et al. 2009; Nov et al. 2014) and conservationists (Ryan et al. 2001; Bruyere and Rappe 2007; Asah and Blahna 2012; Maund et al. 2020).

In contrast to many other groups, beekeeper citizen scientists have an almost professional, though not necessarily scientific, interest in the investigation subject because of the income they make from hive products. In citizen science, beekeepers can be regarded as experts, as has been shown for other groups of volunteers (Ottinger 2010; Callaghan et al. 2020). Beekeepers volunteering to participate in the INSIGNIA citizen science project were mostly male and older than 55 years. Regarding the question on sex, we wish to note that some of the participants to our knowledge were couples or groups such as a school or a beekeeping association. In further studies, we therefore suggest to include the answer option “group”. The participation of older people was also reported by Alender (2016) for a study on volunteers involved in water monitoring. Even in online citizen science, Nov et al. (2014) reported average age to be above 40 in two, and above 50 in one citizen science investigation (namely the Citizen Weather Observer Program), respectively. Beekeepers participating in our study can be rated as experienced in beekeeping, as shown by the number of colonies they manage (on average 70) and a mean of 20 years of beekeeping experience. Forty-seven percent of the volunteers in the study were college graduates.

We did not compare formal education levels to the demography of the countries where the citizen scientists lived, but a study on Illinois’ RiverWatch citizen scientists suggested an education bias towards higher education (Blake et al. 2020). Toomey and Domroese (2013) further found that mostly citizens with higher education and moderate to higher income volunteered as “Bee Watchers” in the “Great Pollinator Project” in New York City. A similar bias is probably true for employment. In our study, however, approximately one-third each of volunteers were full-time employed, retired or self-employed. The majority of citizen scientists in this study were recruited through personal contact, which may indicate a rather targeted recruitment process by the national coordinators of beekeeping communities with which they were very familiar (Brouwer and Hessels 2019). Note that this result was probably only possible due to the rather low number of beekeepers per country participating in the project; in investigations with more participants, recruiting via personal contact would inevitably be less dominant.

In contrast to the motivation of citizen scientists to participate in online research projects (Raddick et al. 2009; Nov et al. 2014), the reason that beekeepers voluntarily help with their private equipment, colonies and work power has not been investigated. We found that the motivation of beekeepers was similar to previous studies on environmental volunteer motivations with “helping the environment” and “I want to contribute to scientific knowledge” being strong motivators, whereas “advancing one’s career through gained experience and networking” and “enhancing reputation in the community” were the weakest motivators (Ryan et al. 2001; Evans et al. 2005; Bruyere and Rappe 2007; Asah and Blahna 2012; Alender 2016; MacPhail and Colla, 2020). We found few motivation items to be affected by demographic traits. Helping the environment and learning new skills or knowledge were stronger motifs for female participants, whereas advancing the career was less important for the older participants (and also those with more beekeeping experience). The strongest demographic effect on motivation seems to be education. We found differences based on formal education in more than half of the motivation items, and consistently with higher forms of agreement among participants without college education (Table S1). Bieszczad et al. (in prep.) interviewed four citizen scientists during the first year of the INSIGNIA project. They showed that even among the small number of interviewees, a range of rationales for citizen science beekeepers was found. These ranged from citizen scientists seeing themselves as active producers of knowledge, to mere assistance in sample taking. The same study indicated that Austrian beekeepers volunteering in this project also had a history of participating in other projects.

The honey bee is often at the centre of discussions on environmental issues, for example in the debates about pesticide legislation or biodiversity loss (Goulson et al. 2015; Suryanarayanan and Kleinman 2013; Blacquière and van der Steen 2017). We therefore wanted to know for the first time, in which areas the volunteers participating in a citizen science project dealing with such topics of high societal relevance would expect the impact of such a project. Our results suggest that volunteer beekeepers in this particular case expected the INSIGNIA project to be most impactful in environment, beekeeping and science (Table 1). On the other hand, they ranked economy and politics as the area where they expected the project having least impact.

Compared to motivation, appreciation of citizen scientists has been less studied. For example, Alender (2016) found that hand-written cards, personalized emails, a volunteer appreciation event or name recognition in the organization’s newsletter are recognized as meaningful ways of appreciation of participation. In addition to the options surveyed by Alender (2016), we added two particular answer options for citizen scientists participating in the INSIGNIA project: results feedback on their own samples (pollen diversity and pesticide residues). Results feedback is a special case of personal reward, but may apply to many other studies where samples are collected from private property or livestock due usually to the high costs for such testing (Van De Gevel et al. 2020; Bieszczad et al. in prep.). Our results clearly demonstrate that beekeepers favour this reward over any other way of recognition of their work; 97% of citizen scientists in our study supported this (Fig. 3). Interestingly, the free laboratory analysis of samples was only rated as the eighth reason for participating in the citizen science project (Fig. 2). Results feedback on pesticide samples was found to be most important for females and participants aged 46-60 years (compared to younger participants, Table S2), but in the light of the overwhelming agreement with this form of recognition, it is important to note that the statistical differences are based on only one diverging answer from a young (male) participant.

We suggest further investigating the role of such profound feedback as motivation and/or appreciation for citizen scientists. Our results also stress how important verbatim reports of the laboratory findings of their collected samples are for beekeepers. This implies also to discussions on the ownership of investigation findings, for example whether citizen scientists may present the results from their hives to colleagues or to the media. The latter actually includes potential for conflict, as for example pesticide residue analyses are of interest to society, but it is important that results are placed in a proper context. It is important to note that we surveyed the beekeepers before they had received results, as sample transport and laboratory analysis took some time. Next to the two already discussed project-specific personal appreciations for beekeepers, citizen scientists also felt group co-authorship on a scientific publication or name recognition in a scientific publication are meaningful recognitions of their work, which is in strong contrast to the findings of Alender (2016). These differences might be due to the different populations of beekeepers and water quality monitors surveyed. In fact, Bieszczad et al. (in prep.) from their interviews with four Austrian citizen scientists in the first year of the project found possible self-conceptions of beekeeper citizen scientists to be “amateur scientist” or “expert”, which may explain the affinity to receive authorship credits. Recently suggested possibilities for crediting citizen scientists’ contribution are indeed group authorship (Ward-Fear et al. 2020) or mentioning their participation in acknowledgements (Gadermaier et al. 2018); the latter was rated as very or moderately meaningful by 69.6% of respondents. Name recognition on social media was found to be the least meaningful way of recognition for ~35% of citizen scientists in our study, probably because it reveals their identity. We therefore advise against (unasked) disclosure of citizen scientists’ identities.

Beekeepers assessed the tasks they were asked to do as part of their citizen science activity as relatively easy. The overall average was 2.5, which is on the lower half of the ten-part difficulty scale. This is not surprising, as the citizen scientists proved to be experienced beekeepers, finding little difficulty to understand and put the instructions into practice. The main tools they had to use were pollen traps, which are common beekeeping equipment in many countries (Hoover and Ovinge 2018), but some citizen scientists in the project were using them for the first time. The APIStrips, which none would have previously used, were however designed to be very user-friendly (Murcia-Morales et al. 2020). Fittingly, the three tasks rated most difficult had little to do with their primary expertise of beekeeping: giving information on the phenology of flowering plants, followed by picking the best day for sampling, and photo documentation of collected samples. The weakness of beekeeper citizen scientists’ ability, or maybe their self-assessed infirmity, to provide information about the plants flowering in the vicinity of their apiaries was surprising and needs further clarification. The difficulty assessment was not affected by age, beekeeping experience or sex of the citizen scientists. Three of the 21 tasks citizen scientists had to accomplish were rated more difficult by volunteers with a college degree (or higher), compared to those not having such a degree.

The apicultural problems most often reported by citizen scientist beekeepers in our study were typical problems occurring in beekeeping like colony dwindling, supersedure or swarming. These troubles did not harm the scientific outcome of the current citizen science investigation, but it is important to note that these can happen. Citizen scientists reported more serious troubles (colony mortality, broken equipment) rarely or not at all (brood diseases). All of these complications should therefore be taken into account when planning a project, so we recommend preparing contingency plans. The worst case in a scientific investigation often is when an experimental colony needs to be replaced during the project. We believe, however, that in the case of investigations where the colonies are used to monitor the environment, rather than the honey bees themselves, such replacements can be made to continue sampling without harming the outcome of the investigation.

An often cited advantage of citizen science studies is the cost-effective data collection (Pocock et al. 2014). Here we estimate the work power gained through citizen science involvement. Our results suggest an average work time of 62.6 min for one sampling of both test colonies, travel to the colonies and submitting the data via internet form. This is a quite similar time budget spent, compared to that of the water quality monitors surveyed by Alender (2016). Average work time for sampling estimated by beekeepers alone was 29.3 min. Travel time varied greatly between beekeepers, depending on where they had their colonies. From the total time spent for sampling, travel and data submission, we estimated the total workload per beekeeper per complete sampling season in the INSIGNIA project to be 10.4 h. Projecting this to 80 participants in the studied season, this would equal 4.8 person months citizen science workload. Considering that citizen scientists often have short travel times to their apiaries, compared to a researcher visiting all of these places, the travel time saving might be even more striking for public participation in environmental sampling studies. As is emerging in the interviews conducted by Bieszczad et al. (in prep.) during the first year of the INSIGNIA project, it is however important that coordinators need to ensure that they do not make citizen scientists feel like “cheap co-workers”. Nonetheless, INSIGNIA citizen science beekeepers to a high degree reported that they would participate again in a similar investigation, and also recommend participation to fellow beekeepers.

Citizen science in bee research can directly and indirectly improve the well-being of bees in various ways, as pointed out by Shirk et al. (2012) for other environmental or conservation projects. First of all, the scientific project results support the scientific evidence of risk factors for honey bees (Goulson et al. 2015). This can increase awareness and understanding of bee biology and ecology in society (Jordan et al. 2011), promoting conservation and sustainable use of terrestrial ecosystems (Koffler et al. 2021). This is consistent with the manifold values to participants reviewed in MacPhail and Colla (2020), including an increase in scientific literacy. In the case of beekeeper citizen scientists, direct benefits for honey bee health include individual participants’ development of new skill sets (Shirk et al. 2012; Morawetz et al. 2020) or insights from environmental learning (Ballard and Belsky 2010; Toomey and Domroese 2013). Finally, the knowledge of beekeepers was previously used in crowdsourcing studies to identify best practice methods to reduce honey bee colony losses (Jacques et al. 2017; Steinhauer et al. 2020; Kulhanek et al. 2021). El Agrebi et al. (2021) demonstrated the importance of beekeepers’ perception of risks as a mitigation strategy for colony losses among Belgian amateur beekeepers.

## Conclusions

This study for the first time reveals the why (motivation) and the how (with what tasks) of beekeeper participation in scientific research. We also surveyed the best ways of recognition and appreciation for the voluntary work of this specialized group of citizen scientists. Knowing the target group and their conception will help in designing successful citizen science studies and recruiting volunteers. We found that receiving laboratory analysis results of the samples from their colonies is the most meaningful way of appreciation for beekeepers, but not their topmost reason for participation. These instead were rather general motivations already reported in other investigations such as helping science and the environment, or to learn something new. Citizen scientists ranked environment, beekeeping and science as the areas where they expected the investigation, they participated in, to be the most impactful. A citizen scientist beekeeper in this study on average spent circa 10 working hours for sampling throughout a season, but volunteers would to a high degree participate in similar studies, or recommend participation to other beekeepers. Our study suggests that beekeepers have a great potential to help research in many different ways. Further steps for citizen science in bee research should be to create more collaborative or co-created research projects (Bonney et al. 2009b; Koffler et al. 2021), where citizens are also involved in identifying research questions or experimental design.

## Supplementary information

ESM 1 (PDF 121 kb)

ESM 2 (XLSX 37 kb)

ESM 3 (LSS 620 kb)

ESM 4 (PDF 122 kb)

## Data Availability

The following information was supplied regarding data availability: The questionnaire, multilingual Limesurvey file and the survey results (raw data) are available as supplement.
