# Supplementary material for: How and why beekeepers participate in the INSIGNIA citizen science honey bee environmental monitoring project
Source: Environ Sci Pollut Res Int. 2021 Mar 16;28(28):37995–8006. doi: 10.1007/s11356-021-13379-7 (PMC8302492; doi:10.1007/s11356-021-13379-7)
Supplement: Supplementary file 1 — (PDF 121 kb) [file 11356_2021_13379_MOESM1_ESM.pdf]

Supplementary Material S1

Environmental Science and Pollution Research

How and why beekeepers participate in the INSIGNIA citizen science honey bee environmental monitoring project

Kristina Gratzer[1], Robert Brodschneider[1]*

[1]University of Graz, Institute of Biology, Universitätsplatz 2, A-8010 Graz, Austria

*Corresponding author. E-mail address: robert.brodschneider@uni-graz.at

Full Questionnaire for INSIGNIA citizen scientists

1) Choose country
   a. Austria
   b. Belgium
   c. Denmark
   d. France
   e. Greece
   f. Italy
   g. Ireland
   h. Latvia
   i. United Kingdom
   j. Netherlands
   k. Norway

2) Please select your sex
   a. Male
   b. Female
   c. No answer

3) Please type in your age in years (Only an integer value may be entered in this field)?
   a. Empty text field

4) Years of beekeeping experience (Only an integer value may be entered in this field)?
   a. Empty text field

5) Number of honey bee colonies (Only an integer value may be entered in this field)?
   a. Empty text field

6) Employment (Choose one of the following answers):
   a. full-time employment
   b. part-time employment
   c. self-employment
   d. retired

7) Level of Education (Choose one of the following answers):
   a. Trade/technical/vocational training
   b. High school graduate, diploma or the equivalent
   c. Bachelor's degree
   d. Master's degree
   e. Doctorate degree

8) How did you hear of the INSIGNIA project (Choose one/more of the following answers):
   a. Article in beekeeping magazine
   b. Project or other website
   c. Social media

d. Talk at meeting
e. Personal contact with coordinators
f. Friends
g. Other: ___________

9) Please rate your level of agreement with reason for volunteering in the study

	Strongly agree	agree	neutral or undecided	disagree	strongly disagree
I want to help or enhance the environment	☐	☐	☐	☐	☐
I want to help the community	☐	☐	☐	☐	☐
I want to get outside or connect with nature	☐	☐	☐	☐	☐
I want to contribute to scientific knowledge	☐	☐	☐	☐	☐
I want to learn more about honey bees	☐	☐	☐	☐	☐
I want to do something physically active	☐	☐	☐	☐	☐
I want to learn skills or new knowledge	☐	☐	☐	☐	☐
I want to have fun	☐	☐	☐	☐	☐
I want to help the project to do more for less money	☐	☐	☐	☐	☐
I want to engage with other people	☐	☐	☐	☐	☐
I want to enhance my reputation in my community	☐	☐	☐	☐	☐
I want to advance my career through gained experience or networking	☐	☐	☐	☐	☐
I want to increase public safety	☐	☐	☐	☐	☐

I want to receive free laboratory analysis of my samples	☐	☐	☐	☐	☐

10) In which area do you think the research of INSIGNIA will be most impactful?

- All your answers must be different and you must rank in order.
- Double-click or drag-and-drop items in the left list to move them to the right - your highest ranking item should be on the top right, moving through to your lowest ranking item.
- Please select at most 7 answers

Your choices	Your ranking
Innovation<br>Environment<br>Politics →<br>Economy<br>Beekeeping<br>Science<br>Agriculture	

11) Please indicate how meaningful different forms of recognition are for you:

	Very meaningful	Moderately meaningful	neutral or undecided	Not very meaningful	Not meaningful at all
Hand-written card	☐	☐	☐	☐	☐
Volunteer appreciation event	☐	☐	☐	☐	☐
Certificate or token of appreciation	☐	☐	☐	☐	☐
Paraphernalia (stickers, hats, t-shirts from project)	☐	☐	☐	☐	☐
Name recognition in social media	☐	☐	☐	☐	☐
Name recognition in scientific publication	☐	☐	☐	☐	☐
Results feedback on own samples (pollen diversity)	☐	☐	☐	☐	☐

Results feedback on own samples (pesticide residues)	☐	☐	☐	☐	☐
Individual co-authorship on scientific publication	☐	☐	☐	☐	☐
Group co-authorship on scientific publication ("Insignia-beekeepers")	☐	☐	☐	☐	☐

12) Please indicate the difficulty of the individual tasks of the INSGNIA study (1= very easy, 10= very difficult)

- Only numbers may be entered in these fields.
- Each answer must be between 1 and 10

	Indicate the difficulty of the individual tasks of the INSGNIA study (1= very easy, 10= very difficult)
Communication with coordinators	
Understanding terminology	
Understanding pamphlet	
Understanding study aims	
Prepare colonies for the study	
Organize the materials I needed for the study in time	
Pick best day for sampling	
Pollen trap usage	
Pollen harvest	
Measuring the required amount of pollen	
Working with the APIStrips	
Filling out sample labels	
Estimating number of occupied beelanes	
Giving information on the phenology	
Answering the electronic survey	
Providing location using the online map	
Sample storage	
Test hive management throughout the season	
Retain motivation for participation throughout season	
Photo-documentation	
Accurate working while sampling	

13) Did you experience one of the following complications in your test colonies during 2020 study?

	No	Yes, in one colony	Yes, in both colonies
Supersedure	☐	☐	☐
Swarming	☐	☐	☐
Colony dwindling	☐	☐	☐
Colony mortality	☐	☐	☐
Brood disease (American foulbrood etc..)	☐	☐	☐
Had to replace test colony with another one	☐	☐	☐
Broken Equipment (pollen trap...)	☐	☐	☐

14) Estimate…
(Each answer must be at least 1; Only integer values may be entered in these fields.)

a. Estimate your field working time for one complete sampling of both colonies, not including travel (in minutes)
    a. Empty text field.

b. Please also estimate travel time in minutes (outward and return)
    a. Empty text field.

15) Would you recommend colleagues to participate in similar citizen science studies (Choose one of the following answers)?
a. Definitely
b. Very Probably
c. Probably
d. Possibly
e. Probably Not
f. Definitely Not

16) Would you participate again in a similar citizen science study (Choose one of the following answers)?
a. Definitely
b. Very Probably
c. Probably
d. Possibly
e. Probably Not
f. Definitely Not
