# Supplementary material for: How and why beekeepers participate in the INSIGNIA citizen science honey bee environmental monitoring project
Source: Environ Sci Pollut Res Int. 2021 Mar 16;28(28):37995–8006. doi: 10.1007/s11356-021-13379-7 (PMC8302492; doi:10.1007/s11356-021-13379-7)
Supplement: Supplementary file 4 — (PDF 122 kb) [file 11356_2021_13379_MOESM4_ESM.pdf]

Supplementary Material S4 (Table S1-S3)

Environmental Science and Pollution Research

How and why beekeepers participate in the INSIGNIA citizen science honey bee environmental monitoring project

Kristina Gratzer[1], Robert Brodschneider[1]*

[1]University of Graz, Institute of Biology, Universitätsplatz 2, A-8010 Graz, Austria

*Corresponding author. E-mail address: robert.brodschneider@uni-graz.at

Table S1. Differences in responses on level of agreement with reason for participation among demographic groups of citizen scientists. Where Kruskal-Wallis and post-hoc Mann-Whitney U tests with Bonferroni-Holm correction for multiple comparisons was $p<0.05$, p-value (exact, Monte Carlo method) and the direction of the differences is shown, whereas n.s. = not significant.

**Survey item**	**Age**<br>30-45 years: n=15;<br>46-60 years: n=30;<br>60+ years: n=24	**Experience**<br>0-15 years: n=38;<br>15-30 years: n=16;<br>30+ years: n=15	**Gender**<br>Male: n=55;<br>Female: n=13	**Education**<br>No college: n=36;<br>College: n=32
I want to help or enhance the environment	p=0.013,<br>post-hoc n.s.	n.s.	p=0.025, females agree more	n.s.
I want to help the community	n.s.	n.s.	n.s.	n.s.
I want to get outside or connect with nature	n.s.	n.s.	n.s.	p=0.042,<br>No college agree more
I want to contribute to scientific knowledge	n.s.	n.s.	n.s.	n.s.
I want to learn more about honey bees	n.s.	n.s.	n.s.	n.s.
I want to do something physically active	n.s.	n.s.	n.s.	p=0.029,<br>No college agree more
I want to learn skills or new knowledge	n.s.	n.s.	p=0.047, females agree more	n.s.
I want to have fun	n.s.	n.s.	n.s.	p=0.009,<br>No college agree more
I want to help the project to do more for less money	n.s.	n.s.	n.s.	p=0.001,<br>No college agree more
I want to engage with other people	n.s.	n.s.	n.s.	p=0.021,<br>No college agree more
I want to enhance my reputation in my community	n.s.	n.s.	n.s.	n.s.

I want to advance my career through gained experience or networking	p=0.003,<br>30-45 and 46-60 years agree more than<br>60+	p=0.038,<br>0-15 years agree more than 30+ years	n.s.	p=0.047,<br>No college agree more
I want to increase public safety	n.s.	n.s.	n.s.	p=0.002,<br>No college agree more
I want to receive free laboratory analysis of my samples	n.s.	n.s.	n.s.	p=0.001,<br>No college agree more

Table S2. Differences in responses on levels of agreement with different forms of recognition or appreciation among demographic groups of citizen scientists. Where Kruskal-Wallis and post-hoc Mann-Whitney U tests with Bonferroni-Holm correction for multiple comparisons was $p<0.05$, p-value (exact, Monte Carlo method) and the direction of the differences is shown, whereas n.s. = not significant.

**Survey item**	**Age**<br>30-45 years: n=15; 46-60 years: n=30; 60+ years: n=24	**Experience**<br>0-15 years: n=38; 15-30 years: n=16; 30+ years: n=15	**Gender**<br>Male: n=55; Female: n=13	**Education**<br>No college: n=36; College: n=32
Hand-written card	n.s.	n.s.	n.s.	n.s.
Volunteer appreciation event	n.s.	n.s.	n.s.	n.s.
Certificate or token of appreciation	n.s.	n.s.	n.s.	n.s.
Paraphernalia (stickers, hats, t-shirts from project)	n.s.	n.s.	p=0.039, females rated less meaningful	n.s.
Name recognition in social media	n.s.	n.s.	n.s.	n.s.
Name recognition in scientific publication	n.s.	n.s.	n.s.	n.s.
Results feedback on own samples (pollen diversity)	n.s.	n.s.	n.s.	n.s.
Results feedback on own samples (pesticide residues)	p=0.034, 46-60 years agree more than 30-45 years	n.s.	p=0.032, females rated more meaningful	n.s.
Individual co-authorship on scientific publication	n.s.	n.s.	n.s.	n.s.
Group co-authorship on scientific publication ("Insignia-beekeepers")	n.s.	n.s.	n.s.	n.s.

Table S3. Differences in citizen scientist beekeepers' assessment of difficulty of different study tasks among demographic groups of citizen scientists. Where Kruskal-Wallis and post-hoc Mann-Whitney U tests with Bonferroni-Holm correction for multiple comparisons was p<0.05, p-value (exact, Monte Carlo method) and the direction of the differences is shown, whereas n.s. = not significant.

Survey item	**Age**<br>30-45 years: n=15;<br>46-60 years: n=30;<br>60+ years: n=24	**Experience**<br>0-15 years: n=38;<br>15-30 years: n=16;<br>30+ years: n=15	**Gender**<br>Male: n=55;<br>Female: n=13	**Education**<br>No college: n=36;<br>College: n=32
Communication with coordinators	n.s.	n.s.	n.s.	n.s.
Understanding terminology	n.s.	n.s.	n.s.	n.s.
Understanding pamphlet	n.s.	n.s.	n.s.	p=0.020,<br>College rate more difficult
Understanding study aims	n.s.	n.s.	n.s.	n.s.
Prepare colonies for the study	n.s.	n.s.	n.s.	n.s.
Organize the materials I needed for the study in time	n.s.	n.s.	n.s.	n.s.
Pick best day for sampling	n.s.	n.s.	n.s.	n.s.
Pollen trap usage	n.s.	n.s.	n.s.	n.s.
Pollen harvest	n.s.	n.s.	n.s.	n.s.
Measuring the required amount of pollen	n.s.	n.s.	n.s.	n.s.
Working with the APIStrips	n.s.	n.s.	n.s.	p=0.019,<br>College rate more difficult
Filling out sample labels	n.s.	n.s.	n.s.	n.s.

Estimating number of occupied beelanes	n.s.	n.s.	n.s.	n.s.
Giving information on the phenology	n.s.	n.s.	n.s.	n.s.
Answering the electronic survey	n.s.	n.s.	n.s.	n.s.
Providing location using the online map	n.s.	n.s.	n.s.	n.s.
Sample storage	n.s.	n.s.	n.s.	n.s.
Test hive management throughout the season	n.s.	n.s.	n.s.	n.s.
Retain motivation for participation throughout season	n.s.	n.s.	n.s.	p=0.009, College rate more difficult
Photo-documentation	n.s.	n.s.	n.s.	n.s.
Accurate working while sampling	n.s.	n.s.	n.s.	n.s.
